# Effectiveness of Anti-Psychotics and Related Drugs in the Huntington French-Speaking Group Cohort

**DOI:** 10.1371/journal.pone.0085430

**Published:** 2014-01-15

**Authors:** Gaëlle Désaméricq, Guillaume Dolbeau, Christophe Verny, Perrine Charles, Alexandra Durr, Katia Youssov, Clémence Simonin, Jean-Philippe Azulay, Christine Tranchant, Cyril Goizet, Philippe Damier, Emmanuel Broussolle, Jean-François Demonet, Graca Morgado, Laurent Cleret de Langavant, Isabelle Macquin-Mavier, Anne-Catherine Bachoud-Lévi, Patrick Maison

**Affiliations:** 1 Equipe 01, U955, Inserm, Créteil, France; 2 Faculté de médecine, Université Paris Est, Créteil, France; 3 Service de Pharmacologie Clinique, Hôpital H. Mondor – A. Chenevier, AP-HP, Créteil, France; 4 Département d'Etudes Cognitives, Ecole Normale Supérieure, Paris, France; 5 Unité de recherche clinique, Hôpital H. Mondor – A. Chenevier, AP-HP, Créteil, France; 6 Centre de référence des maladies neurogénétiques, service de neurologie, CHU d'Angers, Angers, France; 7 UMR CNRS 6214 - INSERM U1083, Angers, France; 8 Centre de référence Maladie de Huntington, Hôpital H. Mondor – A. Chenevier, AP-HP, Créteil, France; 9 Département de génétique, Hôpital de la salpêtrière, AP-HP, Paris, France; 10 Departement of Neurology and Movement Disorders, CHRU Lille, Lille, France; 11 UMR837 INSERM – JPArc Team 6, Lille, France; 12 University Lille 2/Law & Health, Lille, France; 13 Service de Neurologie et pathologie du mouvement, Hôpital de la Timone, Marseille, France; 14 Service de Neurologie, CHU Hautepierre, Strasbourg, France; 15 Université de Strasbourg, Strasbourg, France; 16 Université Bordeaux Segalen, Laboratoire Maladies Rares: Génétique et Métabolisme (MRGM), EA4576, CHU Bordeaux, Service de Génétique médicale, Bordeaux, France; 17 Centre d'Investigation Clinique 004, Inserm, Nantes, France; 18 Faculté de Médecine et de Maïeutique Lyon Sud Charles Mérieux, Université Lyon I, Lyon, France; 19 Service de Neurologie C, Hôpital Neurologique Pierre Wertheimer, Hospices Civils de Lyon, Lyon, France; 20 Centre de Neurosciences Cognitives, UMR5229, CNRS, Bron, France; 21 Centre Leenaards de la Mémoire, Département des Neurosciences Cliniques, CHUV, Lausanne, Switzerland; 22 Centre d'Investigation Clinique 006, Inserm, Créteil, France; 23 Pôle Recherche clinique Santé Publique, Hôpital H. Mondor – A. Chenevier, AP-HP, Créteil, France; Charité-Universitätsmedizin Berlin, Germany

## Abstract

*Purpose: * Huntington's disease is a rare condition. Patients are commonly treated with antipsychotics and tetrabenazine. The evidence of their effect on disease progression is limited and no comparative study between these drugs has been conducted. We therefore compared the effectiveness of antipsychotics on disease progression.

*Methods: * 956 patients from the Huntington French Speaking Group were followed for up to 8 years between 2002 and 2010. The effectiveness of treatments was assessed using Unified Huntington's Disease Rating Scale (UHDRS) scores and then compared using a mixed model adjusted on a multiple propensity score.

*Results: * 63% of patients were treated with antipsychotics during the survey period. The most commonly prescribed medications were dibenzodiazepines (38%), risperidone (13%), tetrabenazine (12%) and benzamides (12%). There was no difference between treatments on the motor and behavioural declines observed, after taking the patient profiles at the start of the drug prescription into account. In contrast, the functional decline was lower in the dibenzodiazepine group than the other antipsychotic groups (Total Functional Capacity: 0.41±0.17 units per year *vs.* risperidone and 0.54±0.19 *vs.* tetrabenazine, both p<0.05). Benzamides were less effective than other antipsychotics on cognitive evolution (Stroop interference, Stroop color and Literal fluency: p<0.05).

*Conclusions: * Antipsychotics are widely used to treat patients with Huntington's disease. Although differences in motor or behavioural profiles between patients according to the antipsychotics used were small, there were differences in drug effectiveness on the evolution of functional and cognitive scores.

## Introduction

Drug prescription is a complex process that takes into account many factors: primary clinical data, patient preferences, the prescriber's clinical and personal experience, external rules and constraints and scientific evidence [1]. Randomised controlled trials (RCTs) are considered the best practice methodology (the gold standard) to provide evidence about drug efficacy. However, RCTs have important limitations for informing clinical practice and policy decisions about treatments. In particular, it is unclear whether findings can be applied to patients seen in routine practice and RCTs do not make comparisons between several prescription options. Recent statistical tools [2] can now help address these issues by allowing multiple comparisons through cohort studies in “real-world” conditions. Such comparisons are particularly useful when studying rare diseases, as the number of patients can be low and may limit the feasibility of clinical trials.

Huntington's disease (HD) is a rare, autosomal dominant, neurodegenerative disorder resulting from expansion of a CAG repeat within the IT15 huntingtin (*HTT*) gene on chromosome 4p [3]. It is characterised by choreiform movements and progressive dementia, and, in 33% to 76% of cases, psychiatric manifestations (for example depression, apathy or irritability)[4]. Recent reviews of available drug trials and case reports [5]–[7] conclude that the management of HD is poorly documented. Antipsychotics and related drugs (for example tetrabenazine) are most commonly used for the treatment of chorea [8]. The use of antipsychotics and related drugs (APRs) differs between countries: olanzapine is widely prescribed in France and in the United-Kingdom, tiapride in Germany, and haloperidol is more common in Italy [9]. In France, only tetrabenazine and tiapride are approved for chorea. Little is known about the differences between the effects of different antipsychotics on motor abilities, cognitive disorders, psychiatric disturbances or metabolic impairments.

We aimed to describe APR use in HD patients in conditions of routine practice and compare their effectiveness using disease progression scores.

## Materials and Methods

The study, carried out between 2002 and 2010, was based on the cohort from the Huntington French Speaking Network (HFSG, http://www.hdnetwork.org).

### Patients

Patients were recruited at 13 centres in France and Belgium (Angers, Bordeaux, Bruxelles, Caen, Creteil, Lille, Lyon, Marseille, Nantes, Paris, Rennes, Strasbourg, and Toulouse); 956 patients, all genetically confirmed (CAG ≥37 repeats), completed at least one Unified Huntington's Disease Rating Scale (UHDRS) assessment. Patients were followed for up to 8 years between 2002 and 2010; the mean follow-up was 28.2 months (Table 1). For more than half of the patients, motor symptoms were the initial symptom of the disease. At the time of their first admission in the Huntington French-Speaking Network, 408 of the HD patients (44%) were in Stage 1, 276 (30%) were in Stage 2, 182 (19%) were in Stage 3, 53 (6%) were in Stage 4, and 12 (1%) were in Stage 5 (the final stage) of the disease as characterised by their Total Functional Capacity (TFC) [10].

**Table 1:** Characteristics and evolution of the overall cohort and the groups of patients taking antipsychotics and related drugs.

	Overall			Subjects with anti-psychotics and related drugs			Subjects having never taking anti-psychotics and related drugs		
	n	Baseline	Change per year	n	Baseline	Change per year	n	Baseline	Change per year
Age at HD onset, years	850	43.8±12.0		518	44.7±12.0		283	42.2±12.3	
Male	951	51		546	55		351	45	
CAG repeat length	956	44.8±4.1		548	44.7±4.0		354	45.1±4.4	
**Motor score**	890	35.1±22.4	4.4±0.2	524	43.5±22.2	4.8±0.2	319	28.4±23.4	3.1±0.4
Chorea	889	8.4±6.1	0.4±0.1	524	9.9±6.3	0.5±0.1	319	7.0±6.3	0.6±0.1
Dystonia	887	3.1±3.6	0.7±0.1	524	4.2±4.2	0.9±0.1	318	2.7±4.0	0.5±0.1
**Behavioural score**	885	16.7±11.7	−0.1±0.1	524	18.8±12.0	−0.2±0.2	332	12.9±10.4	−0.3±0.2
**Functional scores**									
Functional assessment scale	927	30.8±6.2	1.5±0.1	538	33.4±6.4	1.8±0.1	337	28.8±5.5	0.8±0.1
Independence scale	928	81.3±16.8	−3.7±0.2	536	73.9±16.8	−4.2±0.3	338	86.8±15.4	−2.0±0.3
Total functional capacity	931	8.9±3.6	−0.8±0.0	539	7.2±3.5	−0.7±0.3	342	10.1±3.3	−0.4±0.1
**Cognitive score**									
Literacy fluency	623	20.4±13.1	−0.7±0.1	341	16.5±11.6	−1.0±0.1	254	24.9±14.4	0.6±0.3
SDMT	590	23.8±14.3	−2.0±0.1	315	17.0±10.9	−1.9±0.1	246	29.6±14.8	−1.3±0.3
Stroop colour	651	44.0±18.3	−2.6±0.2	345	36.5±16.5	−2.7±0.2	257	50.6±19.8	−1.8±0.3
Stroop word	649	60.7±24.0	−4.1±0.2	344	50.3±22.6	−4.2±0.3	256	67.7±24.7	−2.9±0.4
Stroop interference	644	23.5±13.0	−1.4±0.1	339	18.7±11.2	−1.5±0.1	254	28.1±14.4	−0.8±0.2

HD Huntington' disease; SDMT Symbol digit modality test.

n: available data; Baseline: Mean ± SD; Change per year: Mean ± SE.

### Assessments

Patient demographics, age at onset of HD, expanded CAG repeat and body mass index were recorded. The motor, cognitive, behavioural and functional capabilities of each patient were assessed annually using the UHDRS [11]. The motor score quantified 15 different motor signs, with higher scores indicating more severely impaired motor function. The cognitive assessment was composed of three standardised tests: the Stroop interference test, Symbol Digit Modalities Test (SDMT), and the verbal fluency test. The behaviour score measured 11 characteristics; the frequency and severity of each of these were multiplied to give a single score for each characteristic and then added together to give the total behaviour score. The functional assessment tested common daily tasks using three measures: the Total Functional Capacity (TFC), the Independence Scale and the Functional Assessment Scale. All medications and indications for their use were recorded each time the patient was assessed, as well as the time since last evaluation. The average time between first visit and prescription of a drug was 13 months. We focused on antipsychotics (N05A) and the related drug tetrabenazine (N07XX06) as classified according to the Anatomical Therapeutic Chemical (ATC) Classification System [12]. These were chosen as they were the most commonly prescribed APRs within the cohort. The period of exposure to a drug was defined as the time between the date of prescription and the acknowledged end of prescription. All data were collected from electronic case report forms.

### Statistical methods

Descriptive statistics are presented as means and standard deviation (for quantitative variables) or frequency counts by category (for qualitative variables).

We describe the baseline characteristics of the patient at the time of the first prescription of the drug. These were compared by univariate analysis using Chi^2^ tests or Fisher's exact tests (for qualitative variables) and Kruskal-Wallis tests (for quantitative variables). If a patient received more than one drug at the same time or during follow-up, each period of drug prescription was taken into account independently.

We used a mixed model, adjusted with a multiple propensity score, to compare the effects of treatments on UHDRS scores [13]. Mixed models allow data from subjects tested once and data from those who participated in multiple evaluations to be evaluated together. Thus, any bias due to missing data, although not completely eliminated, is minimized. Multiple propensity scores take into account the lack of randomisation; this lack of randomisation is inherent in the observational data due to differences in treatment assignment by physicians and the conditional probability of being treated based on patient profile at the time of the first prescription. Firstly, multiple propensity scores were estimated for each subject and each drug using a multinomial regression model. Baseline characteristics were included if they had an independent association with the prescription of a drug (*P*<0.25). The size of centre (either fewer or more than 100 participants) was also included. Secondly, we estimated causal effects. These included: fixed effects of treatment levels, time from the start of the treatment until the next UHDRS score was taken, the treatment x time interaction and propensity score, and a random effect for the subject/treatment-specific intercepts. We calculated the overall change in UHDRS score over time and compared the effectiveness of treatments.

Analyses were performed using SAS 9.00 (SAS Institute Inc, Cary, North Carolina). All *P* values were two-tailed, and statistical significance was defined as *P*<0.05 for all tests.

### Ethics statement

All patients, whose data are included in the database of the Huntington French-speaking Group cohort, carefully read an information sheet that present the goal of the network and provide their verbal consent to participate the day at the first evaluation. In France, as the study was observational and no biological sample was collected, the data do not justify specific patient consent. The study was approved by the local ethics committee (Comité de Protection des Personnes Ile-de-France IX, Créteil, France).

## Results

### Prescription of APRs

**Figure 1:**
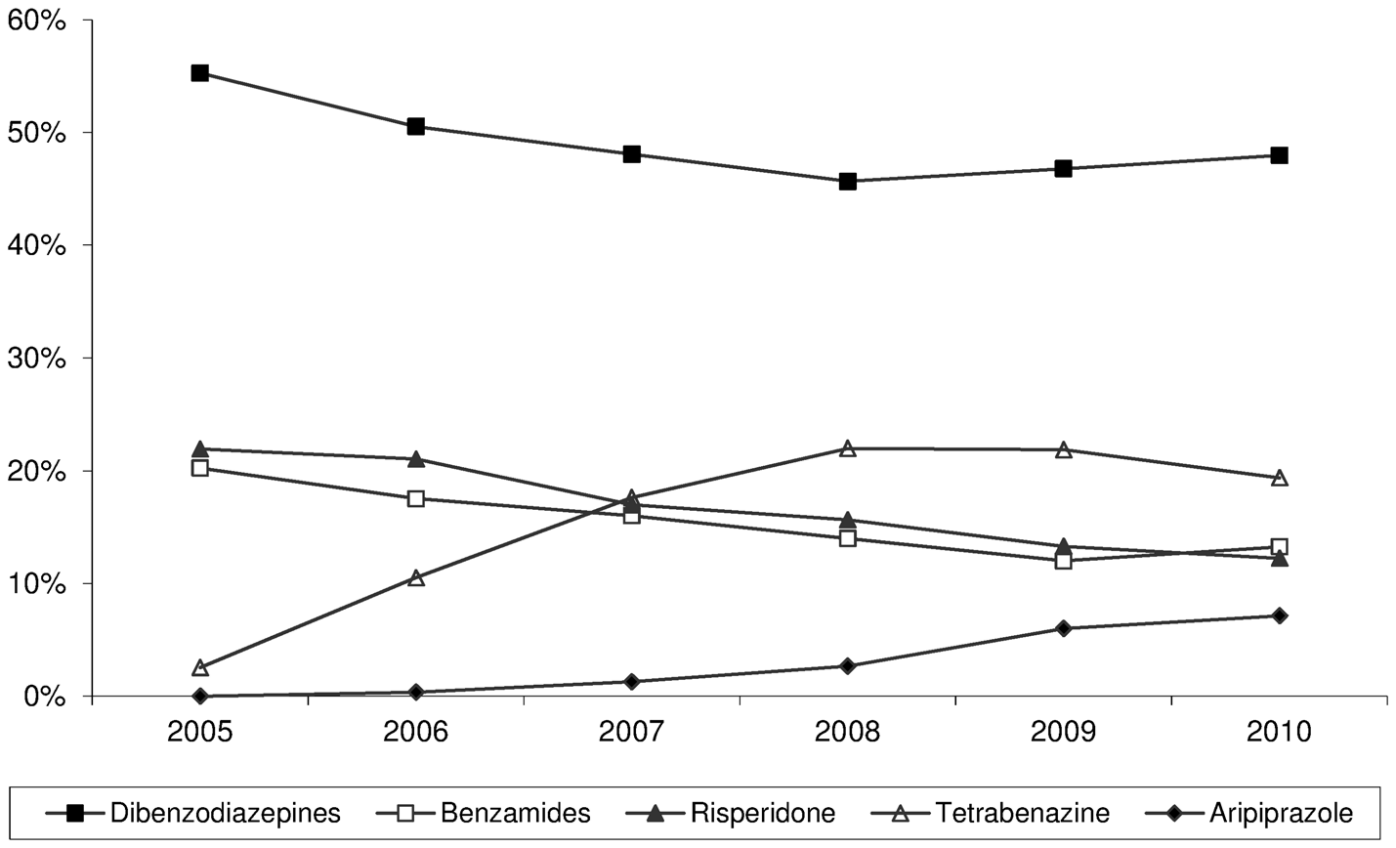
Percentage of prescriptions made for each class of antipsychotic and related drugs (2002–2010). Aripiprazole and tetrabenazine obtained market authorisation in 2004 and 2005, respectively.

During the study period, 602 patients (63% of the overall HD population) received APRs. The most commonly prescribed medications were dibenzodiazepines (38%, mainly olanzapine), risperidone (13%), tetrabenazine (12%) and benzamides (12%, mainly tiapride). Sixty-four percent of these patients were using APRs because of chorea and the others for behavioural disorders such as irritability, aggressiveness or agitation. Figure 1 shows the percentage of prescriptions for each class of drug between 2002 and 2010. As only a small number of patients were treated with aripiprazole, this medication was not taken into account in the following analyses.

### Characteristics of the treated population at the first prescription

A total of 347 patients treated with APRs were evaluated just before the beginning of their treatment. Patients treated with dibenzodiazepines had a shorter history of HD at baseline whereas neither age nor age at disease onset differed between patients treated with the other APRs (Table 2). Approximately half of all patients were also treated with an antidepressant. At the initiation of APR treatment, patients taking dibenzodiazepines tended to have less severe motor impairments than those taking other treatment (*P* = 0.05); there was no significant difference in motor score, except in eye movement scores for which *P* = 0.006. Behavioural scores were similar between APR groups. Scores on the Functional Assessment Scale (FAS), Independence Scale (IS) and Total Functional Capacity (TFC) differed between the four treatment groups. Patients taking dibenzodiazepines were less disabled than others; they had lower FAS scores (32.4±6.3) and the highest IS (75.7±17.2) and TFC (7.8±3.4) scores. The four APR treatment groups performed differently in the verbal fluency test (p<0.05) and the Stroop interference test (p<0.05). Dibenzodiazepines were prescribed to patients with less impairment as defined by these tests.

**Table 2:** Baseline patient profile on the day of first prescription of an anti-psychotic or related drug.

	Dibenzodiazepines		Risperidone		Tetrabenazine		Benzamides		p
Age (year)	187	49.6±11.6	60	52.1±10.1	63	53.1±12.4	56	49.7±10.5	NS
Age at HD onset (year)	179	42.9±12.0	55	44.0±10.1	61	44.9±12.3	52	42.0±10.4	NS
Males (%)	186	52.2	60	48.3	63	49.2	56	60.7	NS
Study year	175	11.6±3.4	54	10.8±3.2	58	11.6±3.3	54	11.9±3.4	NS
Duration of HD (year)	179	6.9±5.5	55	8.5±5.0	61	8.5±4.7	52	8.0±4.6	0.002
CAG repeat length	187	45.3±4.1	60	44.0±3.4	63	44.5±3.3	56	45.6±4.2	0.05
Antidepressant use (%)	187	49.2	60	53.3	63	65.1	56	44.6	NS
BMI	153	22.8±3.7	51	23.2±4.5	44	22.2±3.5	45	23.2±3.7	NS
**Motor score**	177	44.3±21.7	58	45.9±20.6	57	50.6±22.0	51	52.3±24.0	0.05
**Behavioural score**	176	20.0±11.9	56	22.5±10.3	59	18.0±12.6	50	20.9±11.7	NS
**Functional scores**									
Functional Assessment Scale	182	32.4±6.3	59	34.7±6.5	61	34.8±7.2	54	36.4±7.5	0.0005
Independence scale	181	75.6±17.2	59	70.6±16.3	60	69.0±18.6	53	66.5±18.9	0.0003
Total Functional Capacity	182	7.8±3.4	59	6.6±3.2	62	6.8±3.3	55	6.2±3.8	0.004
**Cognitive scores**									
Literacy fluency 1mn	113	18.9±11.3	39	14.1±11.0	42	16.8±12.1	39	13.9±8.1	0.01
SDMT	108	18.7±10.7	33	13.4±8.9	40	15.5±11.6	38	15.7±9.8	NS
Stroop colour	112	38.6±15.8	38	31.2±16.6	42	32.4±15.6	40	32.1±14.0	NS
Stroop word	112	51.7±20.7	38	44.7±23.0	42	47.3±20.5	40	43.5±21.2	NS
Stroop interference	112	20.5±11.1	37	14.3±10.0	42	15.9±12.0	40	16.0±11.5	0.007

SDMT: Symbol digit modality test, BMI: body mass index.

Mean ± SD.

### Impact of these APRs on disease evolution

No significant differences were observed between treatments in terms of motor or behavioural scores (table 3). There was no significant difference between treatment groups for changes in score for chorea (dibenzodiazepines: 0.3±0.2 units per year, risperidone: 0.4±0.4 units per year, tetrabenazine: 0.8±0.5 units per year, benzamides: 0.2±0.5 units per year), and for dystonia impairment (dibenzodiazepines: 0.8±0.1 units per year, risperidone: 0.9±0.3 units per year, tetrabenazine: 1.0±0.4 units per year, benzamides: 1.1±0.3 units per year) but for total motor score minus involuntary movement scores, dibenzodiazepines and risperidone performed better than tetrabenazine (dibenzodiazepines: 2.2±0.2 units per year, risperidone: 2.4±0.4 units per year, tetrabenazine: 4.0±0.6 units per year, benzamides: 3.3±0.5 units per year). There was no significant difference between treatment groups for changes in score for apathy (dibenzodiazepines: 0.4±0.2 units per year, risperidone: 0.5±0.3 units per year, tetrabenazine: 1.0±0.4 units per year, benzamides: 0.4±0.4 units per year), obsessive-compulsive symptoms (dibenzodiazepines: 0.6±0.2 units per year, risperidone: 0.6±0.5 units per year, tetrabenazine: 0.0±0.6 units per year, benzamides: 0.0±0.6 units per year), symptoms of depression (dibenzodiazepines: −0.7±0.4 units per year, risperidone: −0.7±0.8 units per year, tetrabenazine: −0.7±0.9 units per year, benzamides: −0.5±0.9 units per year) or psychosis (dibenzodiazepines: 0.0±0.1 units per year, risperidone: 0.0±0.1 units per year, tetrabenazine: 0.1±0.1 units per year, benzamides: 0.0±0.1 units per year). For irritability and aggression, dibenzodiazepines and tetrabenazine performed better than risperidone (dibenzodiazepines: −0.5±0.2 units per year, risperidone: 0.6±0.4 units per year, tetrabenazine: −0.9±0.5 units per year, benzamides: −0.6±0.5 units per year). An adjustment on multiple propensity score and antidepressant use over time did not modify the results for behavioral scores.

**Table 3:** Pairwise comparisons of effectiveness during the treatment period.

		Dibenzodiazepinesseses	Risperidone	Tetrabenazine
**Motor score** (n = 305)	Risperidone	0.05±1.06		
	Tetrabenazine	−2.15±1.29	−2.21±1.53	
	Benzamides	−1.77±1.21	−1.82±1.46	0.39±1.64
**Behavioural score** (n = 305)	Risperidone	−0.65±0.93		
	Tetrabenazine	−1.10±1.04	0.55±1.26	
	Benzamides	0.52±1.06	1.17±1.28	0.62±1.36
**Functional scores**				
Functional Assessment Scale (n = 301)	Risperidone	−0.55±0.33		
	Tetrabenazine	−0.09±0.38	0.45±0.46	
	Benzamides	−0.61±0.36	−0.06±0.44	−0.51±0.48
Independence scale (n = 297)	Risperidone	−0.16±0.85		
	Tetrabenazine	−1.10±0.97	−0.93±1.18	
	Benzamides	−0.17±0.92	0.00±1.14	0.93±1.23
Total Functional Capacity (n = 301)	Risperidone	−0.41±0.17 * †		
	Tetrabenazine	−0.54±0.19 *	−0.13±0.24	
	Benzamides	−0.31±0.19	0.10±0.23	0.23±0.25
**Cognitive scores**				
Literacy fluency 1 mn (n = 210)	Risperidone	0.34±0.74		
	Tetrabenazine	1.19±0.74	0.84±0.95	
	Benzamides	−0.79±0.74	−1.13±0.95	−1.97±0.94 *
SDMT (n = 201)	Risperidone	0.67±0.57		
	Tetrabenazine	0.06±0.55	−0.61±0.72	
	Benzamides	−1.02±0.49 *	−1.70±0.68 *	−1.08±0.67
Stroop colour (n = 201)	Risperidone	0.22±0.93		
	Tetrabenazine	0.47±0.92	0.24±1.20	
	Benzamides	−1.99±0.85 *	−2.21±1.15	−2.46±1.14 *
Stroop word (n = 206)	Risperidone	0.49±1.38		
	Tetrabenazine	0.84±1.36	0.35±1.78	
	Benzamides	−1.80±1.27	−2.29±1.70	−2.63±1.69
Stroop interference (n = 205)	Risperidone	0.03±0.73		
	Tetrabenazine	0.63±0.71	0.60±0.94	
	Benzamides	−1.42±0.66 *	−1.45±0.90	−2.05±0.88 *

Adjusted difference (line minus column) in mean change per year ± standard error.

SDMT: Symbol digit modality test.

*P<0.05.

† Scores were reversed if necessary (change under risperidone minus the change under dibenzodiazepines  =  −0.41); the mean evolution of TFC was greater with dibenzodiazepines than with risperidone.

There were, however, significant differences for functional impairment: patients taking dibenzodiazepine showed a greater change in TFC score than patients receiving risperidone or tetrabenazine (p<0.05).

Overall, cognitive abilities decreased to a greater extent with benzamide treatment than with dibenzodiazepine, risperidone or tetrabenazine treatments (p<0.05). The impairment in cognition was greater with benzamides than with dibenzodiazepines as assessed with the symbol digit modality test, Stroop colour and interference measures (p<0.05). Significant differences in the literacy fluency (1 minute timing), Stroop colour and interference measures were observed between the benzamide group and the tetrabenazine group (p<0.05). Performance in the symbol digit modality test was also significantly poorer in the benzamide group of patients than in the risperidone group (p<0.05).

The change in body mass index (BMI) over time depended on the APR used. BMI change was significantly less with benzamides (−0.9±0.3 kg/m^2^ per year) than with dibenzodiazepines (0.2±0.1 kg/m^2^ per year) or risperidone (0.0±0.2 kg/m^2^ per year).

There was no significant difference between treatments when analysing tiapride as single drug (n = 43 out of 56 benzamide), except for the results of some of the functional and cognitive assessments. Deterioration was less marked in the Functional Assessment Scale for patients on dibenzodiazepines than for those on tiapride (p<0.05) as well as in the Total Functional Capacity in patients on dibenzodiazepines comparing with patients on tiapride (p<0.05). Decline in the Stroop interference score was greater in patients on tiapride than those on dibenzodiazepines or tetrabenazine (p<0.05).

### Changes in control and treatment groups

In a set of secondary analyses, the group, who had never received an APR, was less severely affected than the groups administered antipsychotics, and the propensity scores failed to correct the differences observed at baseline for motor impairments, some of the behavioural items (dystonia, apathy, obsessive-compulsive symptoms and psychosis), the functional assessment scale and the total functional capacity, and some cognitive assessments (literacy fluency and symbol digit modality test). For chorea, behaviour and symptoms of depression, the patterns of changes through time did not differ between the groups. Irritability and aggression progressed faster in the risperidone group than in the controls (0.8±0.4 units per year, p<0.05). There were significant differences for the independence scale: deterioration was less marked for controls than patients. Decline was greater in the benzamide than controls groups at all Stroop scores (p<0.05) and also at Stroop word and interference (p<0.05) in the dibenzodiazepine group. There were significant differences in the changes in BMI between the control group (−0.1±0.1 kg/m^2^ per year) and the dibenzodiazepine group, where the BMI increased, and benzamide group, where the BMI decreased.

## Discussion

We followed 956 patients from 2002 to 2010; of these, 63% were treated with antipsychotics and related drugs (APRs). Dibenzodiazepines were given to patients with relatively less disability on functional and cognitive scales (literal fluency and Stroop interference) at the time of the first prescription. Patient profiles did not differ in other UHDRS components. Taking into account existing differences in patient profiles at the beginning of the treatment as well as antidepressant undertaken, there was no difference in the changes in motor and behavioural symptoms between the patients on different treatments. The decline in functional score was less severe with dibenzodiazepines than with other APRs. Benzamides were associated with more rapid cognitive decline than the other antipsychotics.

The rate of decline of these patients was similar to that seen in other cohorts (TFC: 0.80±0.00 *vs.* 0.63±0.75 units per year for 129 patients [14], 1.4±0.1 units per year for 92 patients with average follow-up of 3.7 years [15], 0.68±1.39 units per year for 42 patients with average follow-up of 1.9 years [16]; motor capacity: 4.40±0.20 *vs.* 4.42±7.30 units per year [16]). This suggests that our results on effectiveness could be generalised to stages 1 to 3 of the disease. Although our study addressed a rare disease, the number of patients and the length of the study provided sufficient statistical power to obtain significant results. The study is novel in that it provides a comparison of different APRs used in treating HD. We were also able to follow patients for much longer than the relatively short follow-up times of clinical trials (4–12 weeks in clinical trials for antipsychotic drugs [5] *vs.* more than two years in our study).

A secondary goal of this study was to compare disease progression in the four groups of treated patients with that in non-medicated patients. Because, never-treated patients were less severely affected, the value of this group for comparisons provided limited information (even if we use multiple propensity score). A further sub-analysis was performed with patients who received APRs after 2006. This was due to possible changes in prescription habits for tetrabenazine, following the new marketing authorisation in 2005. The results support the same conclusions as the full data sample. The mean time lag between first visit and prescription was 13 months. It is possible that a patient's health could deteriorate during this period and that the administration of APRs could have been started without a visit to a participating centre. We conducted a sub-analysis of the patients for who this delay was only six months or less. The findings were consistent with those for the complete data set (data not shown). Drugs within one class could have a different effect and side effect profile, more patients are needed to analyse each drugs separately. Finally, the side effect profile of APRs depends to a large extent on the dose prescribed. Unfortunately, medicated doses were not available in our study.

In our cohort, tetrabenazine and benzamides tended to be given to patients with a more impaired motor score at the time of first prescription. This may be because they are the only APRs to have market authorisation in France for treatment of motor disability and, in particular, chorea. However, there was no difference in motor decline between patients treated with tetrabenazine and those treated with other APRs (0.8±0.5 units per year). This result contrasts with previous studies showing an improvement in chorea under tetrabenazine by a mean of 5.0 units on the UHDRS after 12 weeks [17] and by 4.6 units after 80 weeks in an open-label continuation study [18]. Tetrabenazine might be less effective against motor symptom progression in the long term, and this would explain the discordance between our and previous studies. Less evidence is available for olanzapine: only one open-label study has shown improvement of the motor scale [19] whereas in two other open-label studies, the statistical tests indicated no significant differences [20], [21]. Two trials with tiapride (the most commonly used benzamide) produced contradictory results and used reduced movement count rather than the UHDRS score to assess motor features [22]. To our knowledge, no clinical trials have assessed risperidone in HD.

Our functional assessments with the TFC scale revealed differences between APR groups. In a multicentre, double-blind, controlled trial (TETRA-HD), 84 ambulatory patients with HD were randomised to receive either tetrabenazine (n = 54) or a placebo (n = 30) for 12 weeks. Tetrebenazine was found to have a deleterious effect on functional capacity [17]. A mild improvement in the disability scale score was observed in case studies with risperidone [23] and olanzapine [24]. A small-scale trial comparing tiapride treatment to a placebo found no difference in functional scores [25].

Antipsychotics may act on multiple psychiatric symptoms. The summary of product characteristics for tetrabenazine states that it is not advisable to initiate this treatment for patients suffering from symptoms of depression. Depressed mood, low self-esteem, anxiety and suicidal ideation as assessed with the UHDRS at baseline did not differ between treatment groups. This suggests that tetrabenazine was not only used in patients with less severe depressive symptoms. Our study shows no difference in behavioural scores between patients on different APRs. It provides no evidence for a deleterious effect of tetrabenazine, or a beneficial effect of dibenzodiazepines, on psychiatric symptoms. In contrast with our results, two small open-pilot studies showed a significant improvement in some UHDRS psychiatric sub-scores (depression, anxiety, irritability and obsessions) after olanzapine treatment and in the short term [21] [20]. There are also case reports of successful risperidone use in aggressive HD patients [26].

Although APRs are commonly used in Huntington's disease, only the TETRA-HD study has reported the effect of tetrabenazine on cognition. Stroop word-reading scores were worse in the tetrabenazine than the placebo group [17]. In our study, tetrabenazine was more effective than benzamides on Stroop colour, Stroop interference and verbal fluency tests. As observed in schizophrenia [27], the decline in digit symbol test scores is smaller with dibenzodiazepines than with benzamides. Thus, the effect of APRs on cognition should be considered when prescribing an antipsychotic for patients with HD.

Weight loss is a common feature in Huntington's disease [28]; the impact of APRs on changes in body mass index over time is well known. As expected, patients under benzodiazepines or risperidone did not loss weight. However, these drugs are associated with weight gain in schizophrenia [29].

In conclusion, we observed similar changes in motor and behavioural scores. Patients taking dibenzodiazapine presented fewer declines in functional scores than patients receiving risperidone and tetrabenazine. Benzamides tended to be associated with the greatest cognitive decline. These differences may be explained by differential pharmacological mechanisms. Tetrabenazine is chemically related to antipsychotic drugs but works as a dopamine depletor by inhibiting the central vesicular monoamine transporter [30]. Benzamides are selective for dopaminergic D2 receptors, dibenzodiazepine and risperidone are less selective for dopaminergic receptors and are antagonists of serotoninergic and alpha-adrenergic receptors. The findings of this observational study, which may have been affected by unmeasured or unaccounted factors, need to be confirmed by further controlled studies. Consequently, we have now initiated a multicentre randomised controlled study (Neuro-HD) comparing olanzapine, tetrabenazine and tiapride. The results are expected in three years.
